# RNA-sequencing and mass-spectrometry proteomic time-series analysis of T-cell differentiation identified multiple splice variants models that predicted validated protein biomarkers in inflammatory diseases

**DOI:** 10.3389/fmolb.2022.916128

**Published:** 2022-08-29

**Authors:** Rasmus Magnusson, Olof Rundquist, Min Jung Kim, Sandra Hellberg, Chan Hyun Na, Mikael Benson, David Gomez-Cabrero, Ingrid Kockum, Jesper N. Tegnér, Fredrik Piehl, Maja Jagodic, Johan Mellergård, Claudio Altafini, Jan Ernerudh, Maria C. Jenmalm, Colm E. Nestor, Min-Sik Kim, Mika Gustafsson

**Affiliations:** ^1^ Bioinformatics, Department of Physics, Chemistry and Biology, Linköping University, Linköping, Sweden; ^2^ Department of Applied Chemistry, College of Applied Sciences, Kyung Hee University, Yong-in, South Korea; ^3^ Department of Biomedical and Clinical Sciences, Linköping University, Linköping, Sweden; ^4^ Department of Neurology, Institute for Cell Engineering, Johns Hopkins University School of Medicine, Baltimore, MD, United States; ^5^ Centre for Personalised Medicine, Linköping University, Linköping, Sweden; ^6^ Navarrabiomed, Complejo Hospitalario de Navarra, Universidad Pública de Navarra, IdiSNA, Pamplona, Spain; ^7^ Department of Clinical Neuroscience, Center for Molecular Medicine, Karolinska Institute, Stockholm, Sweden; ^8^ Biological and Environmental Sciences and Engineering Division, Computer, Electrical and Mathematical Sciences and Engineering Division, King Abdullah University of Science and Technology (KAUST), Thuwal, Saudi Arabia; ^9^ Unit of Computational Medicine, Department of Medicine, Solna, Center for Molecular Medicine, Karolinska Institutet, Solna, Sweden; ^10^ Science for Life Laboratory, Solna, Sweden; ^11^ Department of Neurology, Linköping University, Linköping, Sweden; ^12^ Department of Biomedical and Clinical Sciences, Linköping University, Linköping, Sweden; ^13^ Department of Automatic Control, Linköping University, Linköping, Sweden; ^14^ Department of Clinical Immunology and Transfusion Medicine, Linköping University, Linköping, Sweden; ^15^ Department of New Biology, Daegu Gyeongbuk Institute of Science and Technology, Daegu, South Korea

**Keywords:** proteomics, RNA-seq, T-cell differentiation, biomarkers, multiple sclerosis

## Abstract

Profiling of mRNA expression is an important method to identify biomarkers but complicated by limited correlations between mRNA expression and protein abundance. We hypothesised that these correlations could be improved by mathematical models based on measuring splice variants and time delay in protein translation. We characterised time-series of primary human naïve CD4^+^ T cells during early T helper type 1 differentiation with RNA-sequencing and mass-spectrometry proteomics. We performed computational time-series analysis in this system and in two other key human and murine immune cell types. Linear mathematical mixed time delayed splice variant models were used to predict protein abundances, and the models were validated using out-of-sample predictions. Lastly, we re-analysed RNA-seq datasets to evaluate biomarker discovery in five T-cell associated diseases, further validating the findings for multiple sclerosis (MS) and asthma. The new models significantly out-performing models not including the usage of multiple splice variants and time delays, as shown in cross-validation tests. Our mathematical models provided more differentially expressed proteins between patients and controls in all five diseases. Moreover, analysis of these proteins in asthma and MS supported their relevance. One marker, sCD27, was validated in MS using two independent cohorts for evaluating response to treatment and disease prognosis. In summary, our splice variant and time delay models substantially improved the prediction of protein abundance from mRNA expression in three different immune cell types. The models provided valuable biomarker candidates, which were further validated in MS and asthma.

## 1 Introduction

Identifying biomarkers that can be used in clinical routine to diagnose patients, monitor disease and response to treatment is required for more precision-based medicine (Mayeux, 2004; Chase Huizar et al., 2020). The complex etiology behind many diseases, potentially involving multiple genes and proteins across multiple cell types, renders biomarker discovery for most complex diseases challenging (Rifai et al., 2006).

Proteins are regarded as optimal biomarkers as they are often directly connected to patho-physiological processes as well as serving as targets for many therapeutic interventions (Ek et al., 2021). Whereas measuring global protein levels in a clinical setting remains challenging, gene expression profiling can be readily performed on the limited amount of material obtained from most clinical sampling procedures. Combinations of mRNAs can have high diagnostic efficacy in multiple diseases (Gustafsson et al., 2014; Mao et al., 2018; Gawel et al., 2019; Cha et al., 2020). Ideally, mRNA profiling of clinical samples could be used to identify protein biomarkers for diagnoses, subtyping of diseases and evaluating treatment response.

mRNA expression has often been used to determine corresponding protein levels, even though the accuracy of such estimations can be very imprecise (Gygi et al., 1999; Fortelny et al., 2017). Indeed, the correlation between mRNA and protein expression is often poor (Gygi et al., 1999; de Sousa Abreu et al., 2009; Maier et al., 2009; Vogel and Marcotte, 2012; Fortelny et al., 2017), which becomes highly problematic when using mRNA expression as proxy for protein levels. Several strategies have been proposed to circumvent this issue using more dynamic approaches, as compared to steady-state approximations, accounting for example for spatial and temporal variations in both mRNA and protein expression (Liu et al., 2016; Kuchta et al., 2018). The discrepancy between mRNA and protein abundance is also due to several other factors, including but not limited to differences in the rates of translation and degradation between proteins and cell types (Wethmar et al., 2010). The large number of potential transcript isoforms that can be generated from the same gene due to alternative splicing as well as cell type-specific differences in splice variant use represent additional layers of complexity that complicate the correlation between mRNA to protein (Barbosa-Morais et al., 2012; Floor and Doudna, 2016). To our knowledge, leveraging the contribution and dynamics of different splice variants to infer protein abundance remains largely unexplored.

Here, we developed a novel method incorporating time delay and splice variants to improve protein level inference from mRNA expression. To test our approach, we performed RNA-seq and mass spectrometry proteomics analysis during early human T_H_1 differentiation and used a machine learning modelling approach to infer the relationship between mRNA and protein abundance. T_H_ differentiation is an optimal model system to dissect the relationship between mRNA and protein as 1) primary human naïve T_H_ (NT_H_) cells can be isolated with high purity and in large quantity from human blood (ii), all NT_H_ cells are synchronised in the G_1_ phase of the cell cycle, further reducing inter-cell heterogeneity (Sprent and Tough, 1994) and 3) easy access to large quantities of material enabling relative quantification of mRNA and associated protein abundance to be assayed over time (Schmidt et al., 2018). Moreover, T_H_ cells are important regulators of immunity and thereby associated with many complex diseases, and T_H_1 differentiation itself is pathogenetically relevant in several diseases (Raphael et al., 2015). The utilised models were based on a time delayed linear model between mRNA splice variants of the same gene and protein levels. We generalised the model by applying it onto recent data from human regulatory T (T_reg_) cell and murine B cell differentiation. By combining the strength of time-series analysis and RNA-sequencing, we noted a much better agreement between our mRNA-based measures and proteomics. To test our models, we showed the potential clinical usefulness by predicting potential biomarkers in five complex diseases using our derived models. Analysis of these predicted proteins in asthma and multiple sclerosis (MS) supported their biological relevance. Finally, we validated one of the predicted biomarkers, sCD27, using two independent cohorts of MS patients, which showed a remarkably better stratification between patients and controls than any of our previously reported protein biomarkers. The application of our approach to multiple different cell types, species and diseases shows its general applicability to increase the power of mRNA-based studies for biomarker discovery.

## 2 Materials and methods

### 2.1 Isolation of naïve CD4^+^ T helper cells and T_H_1 polarization

**FIGURE 1:**
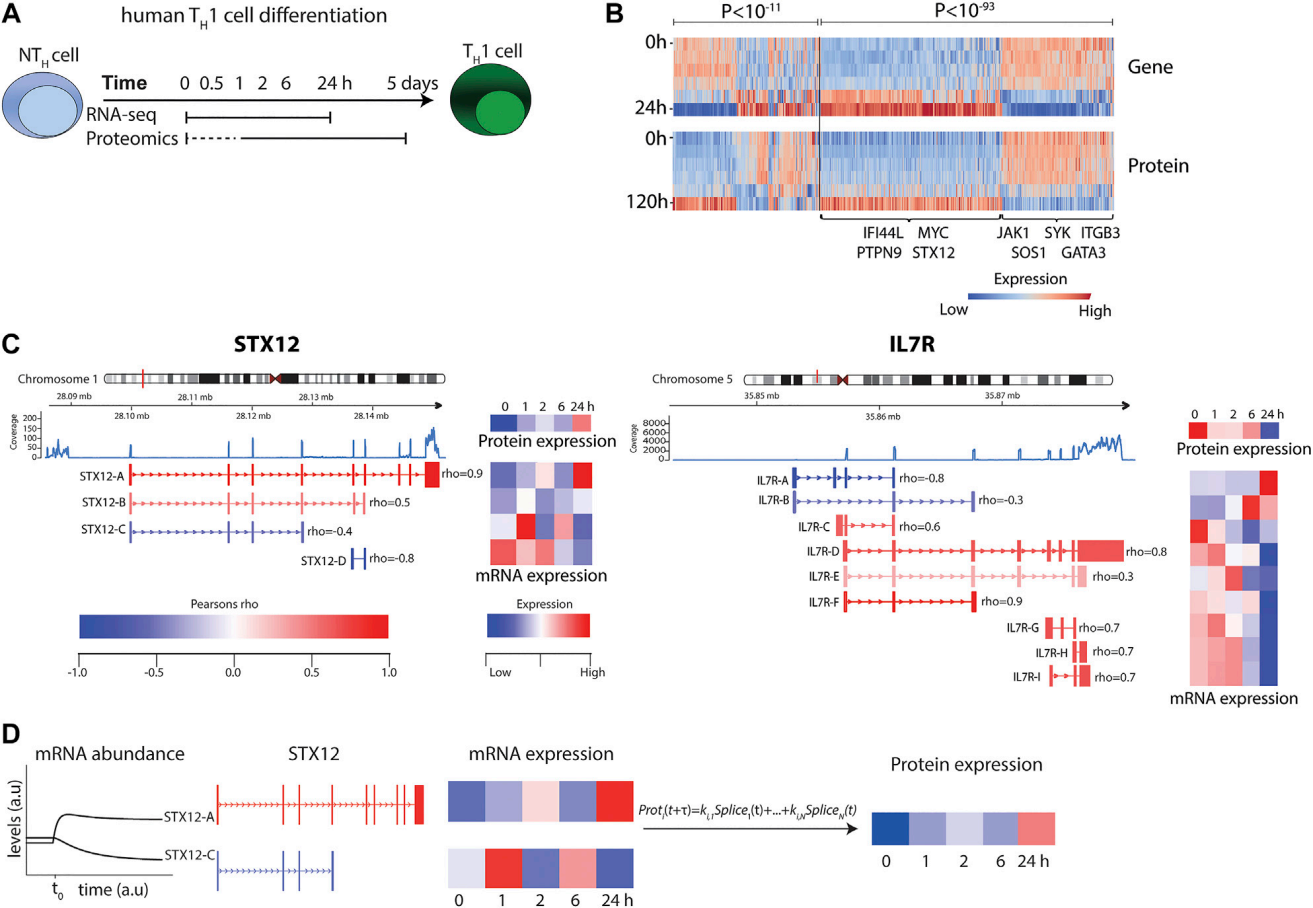
RNA-seq and mass-spectrometry analysis of T_H_1 differentiation revealed highly variable correlations. **(A)** Experimental design. **(B)** Heat map of transcript and protein abundance dynamics in genes that show significant negative (left) and positive (right) correlations. Genes of particular relevance for T cells and T cell differentiation are highlighted in the figure. **(C)** Examples of transcript splice variants showing that both *STX12* (left) and *IL7R* (right) were significantly negatively and positively correlated with protein levels. **(D)** Illustration of the modelling procedure for resolving the poor correlation, using *STX12* as an example.

Peripheral blood mononuclear cells (PBMC) were isolated from blood donor derived buffy coats (*n* = 12), purchased at the blood bank facility at Linköping University Hospital, through gradient centrifugation using Lymphoprep™ (Axis Shields Diagnostics, Dundee, Scotland). Naïve CD45RA^+^ CD4^+^ T cells were isolated with negative immunomagnetic selection using the “Naive CD4^+^ T Cell Isolation Kit II, human” (Miltenyi Biotec, Bergisch Gladbach, Germany) according to the instructions provided by the manufacturer. Cells were suspended in RPMI 1640 media containing L-glutamine, 10%FBS and 1% Penicillin/Streptomycin mixture (all from Gibco, Thermo Fisher Scientific, Waltham, MA, United States) and subsequently activated and polarized towards T_H_1 using Dynabeads™ Human T-Activator CD3/CD28 (1 bead/cell) (Dynal AS, Lillestøm, Norway), 5 ng/μl recombinant human IL-12p70, 10 ng/μl recombinant human IL-2 and 5 μg/μl anti-IL-4 antibodies (clone MAB204; all three from Bio-Techne, Minneapolis, MN, United States). The cells were cultured and differentiated at 37°C, with 5% CO_2_ for 0 min, 0.5, 1, 2, 6 and 24 h for RNA-seq and 0 min, 1, 2, 6, 24 h and 5 days for proteomics (Figure 1A). The earliest time point for the RNA-seq time series was determined based on the change in expression of *IL2, IFNG* and *TBX21* at 3, 5, 10, 15, 30 and 60 min of T_H_1 differentiation, measured by qPCR, where the expression of *IL2* and *IFNG* was significantly increased after 30 and 60 min (*p* < 0.05, Student’s t-test) (See Supplementary Methods and Supplementary Figure S1). After cell culture, the cells were processed for RNA and protein extraction. An overview of the study is shown in Figure 1A and Supplementary Figure S2.

### 2.2 RNA-sequencing

#### 2.2.1 Extraction of RNA

RNA was isolated using the ZR-Duet DNA/RNA kit (Zymo Research, Irvine, CA, United States) following the protocol provided by the manufacturer. The RNA was stored at −80°C until library preparation.

#### 2.2.2 Library preparation and sequencing

The RNA library preparation and subsequent RNA-sequencing (RNA-seq) were carried out by the Beijing Genomics Institute (https://www.bgi.com/global/). Library preparation was performed using the TruSeq RNA Library Prep Kit v2 (Illumina, San Diego, CA, United States). Each sample was sequenced to the depth of 40 million reads per samples with pair end sequencing and a read length of 100 bp on an Illumina 2500 instrument (Illumina).

#### 2.2.3 RNA-seq analysis

All RNA-seq data, both in-house and public, were processed similarly using the following pipeline: Sample qualities were assessed with fastQC (Version 0.11.8) and the mRNA reads were subsequently aligned using STAR (version 2.6.0c) (Dobin et al., 2013), with the parameter “--outSAMstrandField intronMotif” and “--out Filter Intron Motifs Remove Noncanonical,” to the “Homo_sapiens.GRCh37.75.dna. primary_assembly.fa” from Ensemble. The resulting read alignment bam files were assembled into transcripts with StringTie (version 1.3.4d) (Pertea et al., 2015), with default parameters, using the GRCh37.75 gtf annotation from Ensemble. To evaluate mRNA to protein relationship, the mRNA reads were mapped to the mass spectrometry signal of protein abundance using the Homo.sapiens and Mus.musculus package in R (BC., T., 2015a; BC., T., 2015b). Correlations were calculated using Pearson correlations across gene expressions, i.e., one coefficient per gene.

### 2.3 Mass spectrometry

#### 2.3.1 Protein extraction

The cells were thawed and resuspended in 100 μl of 8 M Urea in 40 mM Tris-HCl (pH 7.6) (Sigma-Aldrich, Saint Louis, MO, United States). Ten million cells per time point and biological replicate were pooled from 3–5 samples from different individuals to reach the necessary amount of material required for subsequent analysis steps. In total, cells were isolated from 12 different individuals to achieve the necessary amount of material. The suspension was sonicated using focus sonicator (Sonic Dismembrator 500, Thermo Fisher Scientific, Waltham, MA, United States) for 3 cycles of 10 s pulse with 10 s intervals at 10% of power. After sonication, a magnetic rack was used to remove the T-Activator beads used for the polarization. Protein concentration was measured using the Pierce™ BCA Protein Assay Kit (Thermo Fisher Scientific). 40 ug of each sample were used for digestion.

#### 2.3.2 In solution digestion

Reduction and alkylation of disulfide bonds on proteins were carried out using 1 M dithiothreitol (Roche, Switzerland; final sample concentration 10 mM) for 45 min and 1 M Iodoacetamide (Sigma-Aldrich; final sample concentration 30 mM) for 30 min in a dark, respectively. Following alkylation and reduction, the samples were diluted with ammonium bicarbonate buffer (pH 8.0) until the urea concentration was 1 M (Sigma-Aldrich). The proteins were digested with trypsin (MS grade; Promega, Madison, WI, United States) overnight at 37°C at an enzyme to protein ratio of 1:20. Finally, the peptides were acidified with 100% Trifluoroacetic acid (TFA; Sigma-Aldrich) to a final concentration of 1% TFA and then desalted using macro spin columns (Harvard apparatus, Holliston, MA, United States).

#### 2.3.3 TMT labeling

Peptides were labeled with 6-plex TMT reagent using manufacturer’s protocol with some modification (Thermo Fisher Scientific). The six peptide samples from each time series were resuspended in 100 μl of 100 mM TEAB buffer (pH 8.0; Sigma-Aldrich) and a unit of each TMT reagent was resuspended in 40 μl of acetonitrile. Subsequently, the prepared TMT reagent was transferred to the peptide sample and then vortexed. The samples were incubated for 2 h at room temperature (RT). The labelled peptide samples from each time series were pooled and concentrated by vacuum centrifugation. The labelled sample was resuspended 100 μl with 10 mM ammonium formate (Sigma-Aldrich) in water (pH 10).

#### 2.3.4 High pH fractionation

The TMT labelled samples were separated using an analytical column (Xbridge, Waters, MA, United States; C18, 5 μm, 4.6 mm × 250 mm) on the Agilent 1200 series HPLC system (Agilent Technologies, Santa Clara, CA, United States). Peptides were eluted using following gradient over 115 min: 0–10 min 0% B, 10–20 min 5% B, 20–80 min 35% B, 80–95 min 70% B, 95–105 min 70% B, 105–115 min 0% B; 10 mM ammonium formate (pH 10; Sigma-Aldrich) was mobile phase A, and 10 mM ACN (pH 10) was mobile phase B. The 96 fractions were added up into 24 fractions, vacuum dried and stored at −80°C after desalting.

#### 2.3.5 LC-MS analysis

The fractionated peptides were analysed on an Orbitrap Fusion Lumos Tribrid Mass Spectrometer (Thermo Fisher Scientific) coupled with the Easy-nLC 1200 nano-flow liquid chromatography system (Thermo Fisher Scientific). The peptides from each fraction were reconstituted in 0.1% formic acid and loaded on an Acclaim PepMap100 Nano-Trap Column (100 μm × 2 cm; Thermo Fisher Scientific) packed with 5 μm C18 particles at a flow rate of 5 μl per minute. Peptides were resolved at 250-nl/min flow rate using a linear gradient of 10%–35% solvent B (0.1% formic acid in 95% acetonitrile) over 95 min on an EASY-Spray column (50 cm × 75 µm ID), PepMap RSLC C18 and 2 µm C18 particles (Thermo Fisher Scientific), which was fitted with an EASY-Spray ion source that was operated at a voltage of 2.3 kV. Mass spectrometry analysis was carried out in a data-dependent manner with a full scan in the mass-to-charge ratio (*m/z*) range of 350 to 1,800 in the “Top Speed” setting, 3 seconds per cycle. MS1 and MS2 were acquired for the precursor ions and the peptide fragmentation ions, respectively. MS1 scans were measured at a resolution of 120,000 at an *m/z* of 200. MS2 scan was acquired by fragmenting precursor ions using the higher-energy collisional dissociation method and detected at a mass resolution of 30,000, at an *m/z* of 200. Automatic gain control for MS1 was set to one million ions and for MS2 was set to 0.1 million ions. A maximum ion injection time was set to 50 ms for MS1 and 100 ms for MS2. Higher-energy collisional dissociation was set to 35 for MS2. Precursor isolation window was set to 0.7 *m/z*. Dynamic exclusion was set to 35 s, and singly charged ions were rejected. Internal calibration was carried out using the lock mass.

#### 2.3.6 Peptide and protein identification

The obtained data were analysed using MaxQuant (version 1.6.0.1). MS raw data were searched using Andromeda algorithm with matching to the Uniprot human reference (released in November 2017). A specificity of trypsin was determined at up to 2 missed cleavages. In modification, carbamidomethylation, TMT 6-plex modification at lysine and N-termination were set as the fixed modifications, and oxidation of methionine was set as a variable modification. The false discovery rate (FDR) for peptide level was evaluated to 0.01 for removing false positive data. For highly confident quantifications of protein, protein ratios were calculated from two or more unique quantitative peptides in each replicate. Data was normalized and removed contaminant and razor peptide. To enrich differentially expressed proteins (DEPs), we analysed the quantitative ratios (as the Log2 value). The fold-change ratio cut off was more than 2 or less than 0.5 based on intensity of 0 min. Searched data went through statistical process with Perseus (version 1.5.1.6).

### 2.4 Mathematical modelling

#### 2.4.1 Splice variant model construction

We hypothesized that protein abundance could be predicted using a linear combination of the corresponding splice variants. To predict protein abundance, we used the Sklearn (Pedregosa et al., 2011) implementation of the LASSO (Tibshirani, 1996), an L1-penalized linear regression model.

$$\begin{array}{c} min\\ \beta ,\in Re \end{array}\left\{\frac{1}{N}{\left\|Y-\beta X\right\|}_{2}+\lambda {\left\|\beta \right\|}_{1}\right\}$$

Here, the time series of one protein is denoted the vector Y, and the corresponding time series of the splice variants are denoted by the matrix X. The rate constant for each splice variant is contained in the vector β. Furthermore, the λ parameter regulates the influence of the L1 term and was determined individually for each protein. The λ term was chosen to minimize the prediction error of a leave-one-out cross validation. In the T_H_1 dataset, the time points differed such that the mRNA abundance also had a measurement at t = 30 min, while the protein data instead had a measurement of t = 120 h. For comparison, the protein data for 30 min was interpolated, while the 120 h time point was omitted. The same procedure was performed using the T_reg_ data from (Schmidt et al., 2018) where T_reg_ were induced by either TGF-β, TGF-β and ATRA, or TGF-β and butyrate. Lastly, the same procedure was performed for mice B cells where B cell differentiation was induced by the Ikaros transcription factor (Gomez-Cabrero et al., 2019) (GSE75417).

#### 2.4.2 Time delay analysis

The effect of time delays between mRNA and protein was analysed since this might affect the prediction of protein abundance. First, we considered the T_H_1 data and linearly interpolated between 0 and 24 h for both the mRNA expression and protein abundance data with a quadratically increasing distribution between the time delays. In total, 200 time series were interpolated, such that the difference between the first time points was 43 s, and the difference between the last samples was 15 min. In the updated model, we added a protein specific time delay τ to regulate which time point of splice variant expression should be used. As an example, a τ = 0.5 h would result in splice variant abundance of t = [0, 1, 2, 6, 24 h] predict protein abundance interpolated at t = [0.5, 1.5, 2.5, 6.5, 24.5 h]. Full details on the models can be found in Supplementary Table S1.

$$min\left\{\frac{1}{N}{\left\|Y\left(t+\;\tau \right)-\beta X\left(t\right)\right\|}_{2}+\lambda {\left\|\beta \right\|}_{1}\right\}$$

#### 2.4.3 Cross validation

To select the values of λ and τ, a double cross-validation was performed (Supplementary Figure S3). First, one of the time points of the protein measurements was removed from the set, leaving only 5 data points. Secondly, a leave-one out cross-validation was performed on the remaining 5 time points, giving an estimate of the accuracy of the model approach given a time delay and a lambda value for the penalty term in the Lasso operator. We used the 200-time delays ranging between 0 and 24 h, and a varying set of lambda parameters (increased until all parameters equaled zero). Thirdly, the time delay and penalization that generated the smallest average squared residuals between the second cross-validation and the data were chosen and used to predict the sixth data point from splice variants. Fourth, this double cross-validation procedure was repeated for all 6 data points.

### 2.5 Differential expression analysis

The raw counts of each transcript were z normalized, and, in the case of predicted protein, combined using the transcript-specific coefficient from the linear model. Next, differential expression was analysed using a non-parametric Kruskal-Wallis test as implemented in the SciPy Python package. We used the Benjamini Hochberg false discovery rate (FDR) when accounting for multiple testing.

### 2.6 Disease prediction

Disease relevance of the splice variant models was tested by re-analysis of RNA-seq case and control material of samples containing conventional CD4^+^ T-cells, i.e., CD4^+^ T-cells with all its sub-types. We found T-cell prolymphocytic leukaemia (T-PLL, GSE100882), asthma in obese children (GSE86430), and allergic rhinitis/asthma (GSE75011) studies through a Gene Expression Omnibus (GEO) repository search and MS through collaboration (James et al., 2018). For each of the studies, we used the T_H_1 and T_reg_ derived models on how to combine mRNA splice variants to predict protein abundance. The resulting sets of predicted protein levels were tested for differential expression between patients and controls using a non-parametric Kruskal-Wallis test. We also applied Kruskal-Wallis tests to the individual splice variants that were used by the models. We assessed model effects by measuring the increase in nominally differential expression from model predictions compared to ingoing splice variants into the model. In the study of MS, we performed a specific gene selection and performed FDR correction using the Benjamini Hochberg selection procedure (FDR < 0.05). Using protein data from two of the largest biomarker studies in MS (Huang et al., 2020; Mahler et al., 2020), we compared the protein measurements with our predicted proteins. One study reported 36 out of 92 proteins as significant (Huang et al., 2020) and another study (Mahler et al., 2020) reported the expression of four proteins whereof two were significant. We found that the expression of all our predicted differentially expressed protein agreed with the two studies (9/9 negatively reported from first study and 1/1 negatively and 1/1 positively reported from second study) and the corresponding P-value was calculated as ((92–36)/92)^9^ x (2/4)^2^ = 2.9 × 10^−3^.

### 2.7 Protein validation

#### 2.7.1 Patients and controls

Cerebrospinal fluid (CSF) was collected from a cohort of 41 patients with newly diagnosed clinically isolated syndrome (CIS) or relapsing remitting MS (RRMS) (Supplementary Table S2) that has been described in more detail elsewhere (Håkansson et al., 2018). All patients fulfilled the revised McDonald criteria from 2010 (Polman et al., 2011). The patients were followed, and new samples obtained after one, two and 4 years. Disease activity was assessed using “no evidence of disease activity” (NEDA), defined by no clinical relapses, no sustained EDSS progression and no new T2 or Gadolinium enhancing lesions. 12 patients at the two year- and 7 patients at the 4-year follow-up were classified as NEDA, whereas patients with relapses, brain MRI activity and sustained disease progression were classified as “evidence of disease activity” (EDA; *n* = 27 and n = 32 at two and 4 years, respectively). Two patients did not complete the study (Håkansson et al., 2018). Twenty-three healthy age-and sex-matched blood donors were included as controls. A second cohort of CSF samples from 16 Natalizumab-treated patients with RRMS or secondary progressive MS (SPMS) was also included. CSF samples were obtained (out of a total of ≈70 included patients with RRMS or SPMS) before and after 1 year of treatment with Natalizumab (Supplementary Table S2). This study cohort has been described previously (Mellergård et al., 2010; Mellergård et al., 2013; Gustafsson et al., 2014). All patients were recruited at the Department of Neurology, Linköping, University Hospital Sweden and both patients and controls gave written consent prior to inclusion. The study was approved by The Regional Ethics Committee in Linköping.

#### 2.7.2 Protein measurements

Quantification of sCD27 was performed using the Human Instant ELISA™ kit from eBioscience (Thermo Fischer Scientific) according to the instructions provided by the manufacturer. The optical densities (O.D.) were read at 450 nm with a wavelength correction at 620 nm in a Sunrise™ microplate reader (Tecan, Männedorf, Switzerland). Data acquisition was performed using Magellan™ version 7.1 computer software (Tecan). The lowest detection limit was 0.63 U/ml and values below the detection limit were given half the value of the detection limit. Statistical differences were determined using Mann-Whitney U-test or Wilcoxon matched-pairs signed rank test (Graphpad Prism v7.04, San Diego, CA, United States). Annexin A1, measured by the human Annexin A1 ELISA kit (Abcam, Cambridge, United Kingdom), was undetectable in all analysed samples (*n* = 32, of whom *n* = 16 samples were included before and *n* = 16 after 1 year of treatment with Natalizumab). Multiplex Bead Technology (MILLIPLEX^®^ MAP Kit, Cat. #: HCYTOMAG-60K-01, Merck Millipore, Burlington, MA, United States) was used to measure soluble CD40L according to the manufacturer’s description. The samples were analysed on a Luminex^®^200™ instrument (Invitrogen, Carlsbad, CA, United States) and data was collected using xPONENT 3.1™ (Luminex Corporation, Austin, TX, United States) analysed using the MasterPlex^®^ Reader Fit (MiraiBio Group, Hitachi Solutions America Ltd., San Bruno, CA, United States). The lowest detection limit was 1.6 pg/ml and values below the detection limit were given half the value of the detection limit. sCD40L concentration was below the lowest detection limit in 71 out of 96 samples (74% undetectable) and was therefore considered as undetectable.

## 3 Results

### 3.1 A significant portion of T-cell genes showed diverse correlations between RNA splice variants and proteins

To generate accurate mRNA and protein models, considering the major factors of time delay and splice variant usage, we first developed a model by analysing early T_H_1differentiation. This was done by performing time series transcriptomic (RNA-seq) and proteomics (mass spectrometry) analysis at six different time points, from 30 min to 5 days, during T_H_1 differentiation, whereof five time points were paired between the omics and could be further used to infer correlations between mRNA and protein (Figure 1A and Supplementary Figures S3, S4). We found a total of 15,699 genes and 6,909 proteins to be expressed during early T_H_1 differentiation. Out of the 6,909 expressed proteins, 5,749 could be mapped to genes and out of those, 4,920 were also found to be expressed at the transcriptomic level. As expected, a significant proportion of the 4,920 genes showed a significant positive correlation between mRNA and protein levels (*n* = 407, expected 123 out of 4,920, binomial test *p* < 10^–93^) during T_H_1 cell differentiation. Interestingly, a significant fraction of negatively correlated genes was also observed (*n* = 205, expected 123, *p* < 10^–11^) (Figure 1B and Supplementary Table S1). Notably, the overall median Pearson correlation (rho) between mRNA and protein was only 0.21. Analysis of the distribution of the correlation coefficients revealed significant enrichments of both positive and negative correlations between splice variants and their corresponding proteins (binomial test for enrichment of significant negative correlation *p* < 1.3 × 10^–3^, odds ratio = 1.48) (Figure 1C and Supplementary Figure S5). For example, the known T-cell associated genes, *IL7R* and *STX12* (Kanduri et al., 2015), contained multiple splice variants, of which several were positively or negatively correlated to their corresponding protein levels (Figure 1C). Given the large variation in correlation between different splice variants of a given gene and its corresponding protein, we proceeded to construct predictive splice variant models of protein abundance.

### 3.2 A linear model combining the expressions of multiple splice variant transcripts showed substantially stronger correlations with protein abundance than individual transcripts

**FIGURE 2:**
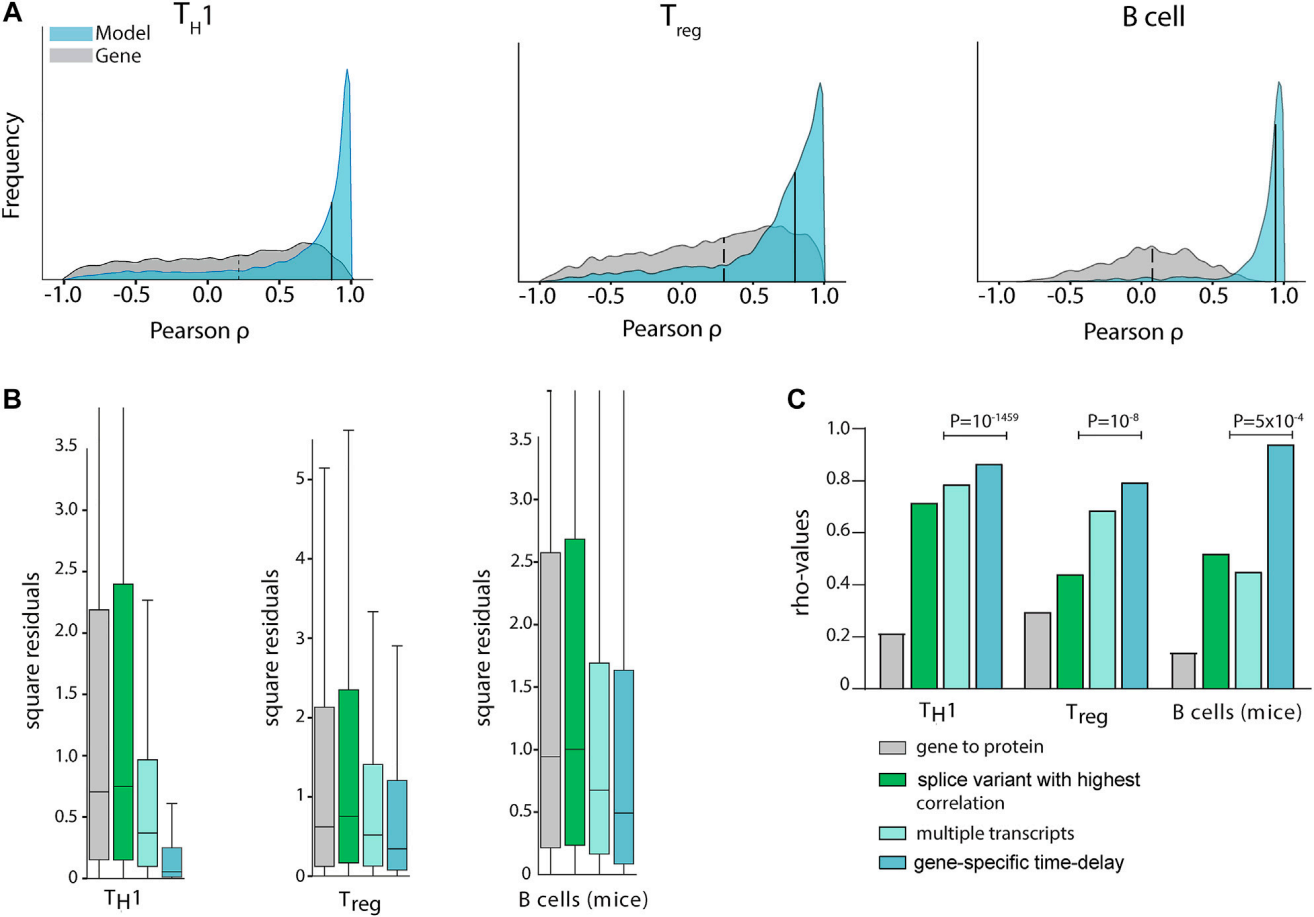
Multiple transcripts and time delays increased mRNA and protein correlations significantly in multiple cell types. **(A)** Gene/protein Pearson correlations in T_H_1 (left), T_reg_ (middle), and murine B-cell (right) differentiation. In the histogram, the grey curve shows the correlation distribution when the sum of all splice variant expressions of a transcript (Fortelny et al., 2017) is used to quantify mRNA abundance (median: dashed line), while in the blue histogram our time delayed multiple splice variant based models are used (medians: solid lines at 0.86, 0.79, and 0.94 for T_H_1, T_reg_ and murine B-cells, respectively). Only cross-validated protein predictions are shown for the proteins for which the null-model could be rejected. **(B)** Out-of-sample cross validation prediction of the three models. Aiming to quantify the predictive power of each added input to the model, we observed that a linear model with gene-specific time delays was the model that generated predictions with the smallest sum of squared residuals. **(C)** Median correlation coefficients (rho) for different mathematical protein prediction models derived from mRNA with increasing protein abundance correlations. P-values were derived from predictions using leave-one-out cross-validation.

In order to construct generally applicable and predictive mRNA-to-protein models, we applied a simple linear relation between the protein abundance of a gene and its associated mRNA splice variants. Furthermore, we allowed for different translation times for each gene. Firstly, we used a cross-validated L1 penalised linear regression model to favour simple models using single splices without any time delays (Figure 1D). The rationale for the L1 penalty was to effectively remove splice variants that carry little or no predictive power over protein abundance. In practice this resulted in maximum of three splice variants per protein for the T_H_1 model, which is a method limitation due to the few data points and our regularisation. This simple model resulted in a median gene-protein correlation of *rho*_TH1_ = 0.86 using cross-validated predictions (Figure 2A). Likewise, to test the generality of the approach we also trained similar models for two existing mRNA-protein time-series datasets with similar results, that is from human T_reg_ cells (Schmidt et al., 2018) (*rho*_Treg_ = 0.79) and mice B cells (Gomez-Cabrero et al., 2019) (GSE75417) (*rho*_Bcell_ = 0.94) (Figure 2A). Next, to test whether the increase in correlation was due to the incorporation of negatively correlating splice variants, multiple transcripts, or time delay, we also constructed such models without each of these parameters. Importantly, our model outperformed the models using only the most highly correlated splice variant for each gene (*rho*_TH1_ = 0.71, *rho*_Treg_ = 0.44, *rho*_Bcell_ = 0.52), and the models using multiple transcripts but without a time delay (*rho*_TH1_ = 0.74, *rho*_Treg_ = 0.69, *rho*_Bcell_ = 0.45) (Figures 2B,C), thus demonstrating that both multiple dynamical splice variants and time delay increase the fit of data and are needed for optimal performance.

To define the optimal time delays between splice variants and proteins, we analysed the time delay distributions and found it to have a mean of 8 h 17 min, 6 h 18 min and 8 h 49 min for T_H_1, T_reg_ and mice B cells, respectively. The detailed parameters of our models are fully displayed in Supplementary Table S1. Next, by using double cross-validation we confirmed that our models could do out-of-sample prediction significantly better than conventional gene expression-based models of protein abundance (binomial test; p_TH1_ = 10^–297^ (expected 14.4 of 28.9, observed 18.0), p_Treg_ = 10^–247^ (expected 21.2 of 43.5, observed 25.2), p_mice B_ = 10^–59^ (expected 2.3 of 5.5, observed 3.3)), and better than static splice variant models which did not include time delays (p_TH1_ = 10^–1459^ (expected 14.8 of 29.6, observed 21.8), p_Treg_ = 10^–8^ (expected 22199 of 44397, observed 22811), p_mice B_ = 5 × 10^–4^ (expected 2.6 of 5.5, observed 2.9), Figure 2C). Moreover, we used time-point scrambling and dynamical correlation analysis to show that our analysis was not seriously affected by time-dependences within the time-series (data not shown). In summary, we have identified simple linear models of mRNA splice variants and time delay which could be used to model the time courses in T- and B-cell differentiation (see the full models in Supplementary Table S1). We would like to emphasize that this is a minimal requirement for mRNA-protein models to be meaningful, so we proceeded to analyse if the models were useful to translational research by identifying biomarkers in complex diseases.

### 3.3 The models showed increased biomarker sensitivity which were further verified in multiple sclerosis and asthma

**FIGURE 3:**
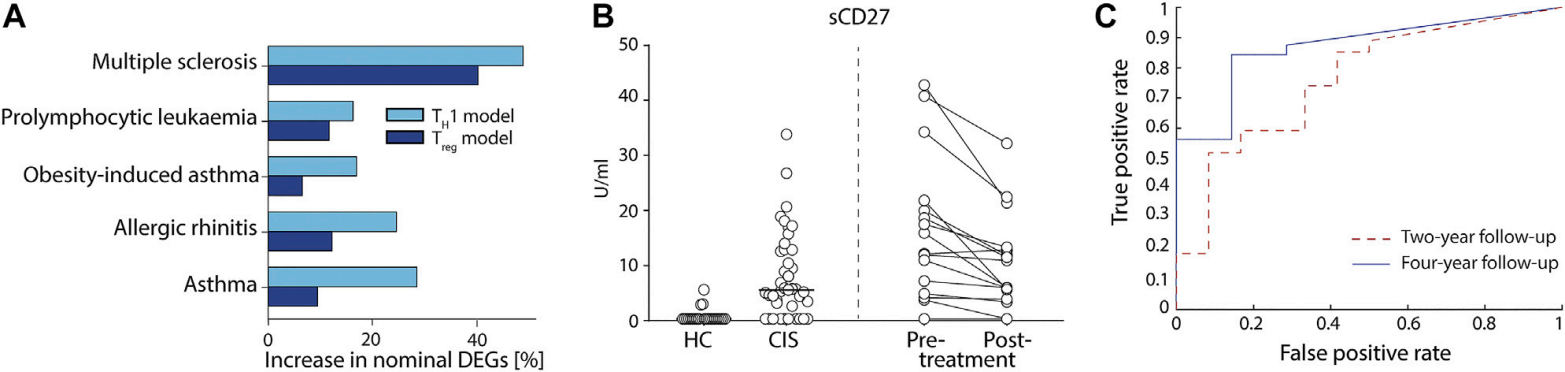
Proteins models led to the discovery of new potential biomarkers of complex diseases that were validated in multiple sclerosis (MS). **(A)** Differential predicted protein (PP) analysis of five diseases using the T_H_1 (light blue) and T_reg_ (dark blue) models showed higher fraction of nominally significant genes than that of normal differential gene expression tests. **(B)** Measurement of actual protein levels of the predicted proteins in a cohort of patients with early MS [clinically isolated syndrome (CIS)] vs. healthy controls (HC) (left side of the figure) and from a cohort of MS patients pre vs. post 1-year treatment with Natalizumab (right side of the figure). sCD27 was measured in cerebrospinal fluid (CSF) using ELISA. **(C)** Receiver operating curve using sCD27 concentration as a single prognostic marker of NEDA at four (solid line) and 2 years (dashed line) after CIS.

Lastly, we aimed to test the potential usefulness of our derived models for the identification of protein biomarkers by applying them on available RNA-seq datasets from human total CD4^+^ T cells. We found datasets for five different diseases (Seumois et al., 2016; James et al., 2018; Johansson et al., 2018; Rastogi et al., 2018); asthma, allergic rhinitis, obesity-induced asthma, pro-lymphocytic leukaemia, and MS, as well as corresponding controls. Because our models correlated well to protein abundances, we hypothesised that differential expression tests using the predicted proteins between patients and controls would be more sensitive than testing directly on the mRNA expression for all splice variants individually. Indeed, we observed that the fraction of nominally differentially expressed genes was higher than using an individual differential expression analysis in all comparisons (binomial *p* < 9.8 × 10^–4^). Moreover, we consistently observed a higher enrichment for the T_H_1 model compared to the T_reg_ model (*p* < 0.03) (Figure 3A), with the highest enrichments in MS and asthma. We therefore proceeded to use our T_H_1 model on MS and asthma.

**FIGURE 4:**
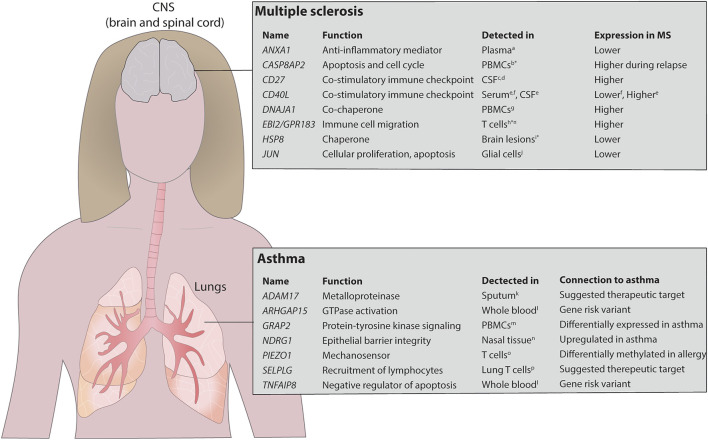
Overview of detected potential biomarkers in asthma and MS. The model identified several proteins that have previously been identified in MS and asthma. The upper panel shows the potential biomarkers identified in MS and the lower panel shows the same in asthma. *mRNA expression, ¤ identified in mice. PBMCs, peripheral blood mononuclear cells. References stated in the figure ^a^Colamatteo A et al., J Immunol, 2019; ^b^Achiron A et al., Ann N Y Acad Sci, 2007; ^c^van der Vuurst de Vries RM et al., JAMA Neurol, 2017; ^d^Wong YYM et al., Mult Scler, 2018; ^e^Masuda H et al., J Neuroimmunol, 2017; ^f^de JG-GJ et al., Immunobiology, 2018; ^g^Bomprezzi R et al., Hum Mol Genet, 2003; ^h^Wanke F et al., Cell Rep, 2017; ^i^Aquino DA et al., J Neuropathol Exp Neurol, 1997; ^j^Bonetti B et al., Am J Pathol, 1999; ^k^Enomoto Y et al., J Allergy Clin Immunol, 2009; ^l^Ferreira MA et al., Nat Genet. 2017; ^m^Persson H et al., J Allergy Clin Immunol, 2015; ^n^Murray JT et al., Biochem J, 2004; ^o^Nestor CE et al., PLoS Genet, 2014; ^p^Purwar R et al., PLoS One, 2011.

First, we compared our MS findings with previously reported proteins using two large biomarker studies (Huang et al., 2020; Mahler et al., 2020) of MS and found a significant agreement comparing our nominal predictions (binomial *p* < 2.9 × 10^–3^; see Methods). Then, we found 20 genes with FDR<0.05, of which none were detected at 20% FDR level by testing for differential expression on the mRNA expression data directly (Supplementary Table S3). Interestingly, eight of the 20 genes had previously been associated with MS (Figure 4 and Supplementary Table S3). To further justify the relevance of the added genes we analysed if CSF levels of these proteins were related to clinical outcome and immunomodulatory treatment in two independent cohorts, newly diagnosed MS patients (clinically isolated syndrome (CIS) and relapsing/remitting MS, *n* = 41) *vs*. healthy controls (HC, *n* = 23), and response to Natalizumab treatment in relapsing remitting MS patients (*n* = 16). In both cohorts, only sCD27 was present in CSF at a detectable level (Supplementary Table S4), while Annexin A1 and sCD40L were not. Analysis of all patients (*n* = 57) *vs*. HC (*n* = 23) showed high separation (AUC = 0.88, non-parametric *p* = 3.0 × 10^–8^, Figure 3B), and treatment with Natalizumab reduced the sCD27 levels by 34% (*p* = 4.9 × 10^–4^). Notably, sCD27 levels at baseline of newly diagnosed MS and CIS patients were able to predict disease activity after 4 years follow up (AUC = 0.87, *p* = 1.2 × 10^–3^, Figure 3C), which was a stronger prediction than that of all our previously reported 14 biomarkers (Håkansson et al., 2018). Taken together, using the splice variants-to-protein model we were able to uniquely identify and validate biomarkers of MS in an independent patient cohort, while these genes could not be discovered using previous state-of-the-art test for differential gene expression.

For asthma we found six of the top 20 genes that were differentially expressed (determined by conventional mRNA expression) to be previously associated with the disease (Supplementary Table S5). Next, we analysed asthma-associated genes uniquely identified by our model and found seven additional genes to be associated with asthma (Supplementary Table S6). Interestingly, these genes had previously also been reported to be relevant for the disease (Enomoto et al., 2009; Nestor et al., 2014; Poole et al., 2014; Dreymueller et al., 2015; Persson et al., 2015; Ferreira et al., 2017), and are currently being evaluated as potential therapeutic targets (Figure 4). Examples of those genes include *NDRG1*, which regulates T_H_2 differentiation, a key driver in asthmatic disease, downstream of the mTORC2 complex (Murray et al., 2004; Heikamp et al., 2014), *ADAM17*, a metalloproteinase involved in lung inflammation (Dreymueller et al., 2015), *PIEZ O 1*, a mechanosensor regulating T cell activation (Liu et al., 2018) and pulmonary inflammatory responses (Solis et al., 2019), and the P-selectin ligand encoding gene *SELPLG*, important for recruitment of lymphocytes to the airways (Leath et al., 2005; Purwar et al., 2011). Furthermore, the immunomodulatory genes *TNFAIP8* and *ARHGAP15* were identified in GWAS studies as shared risk variants for several IgE-mediated diseases including asthma, allergic rhinitis and atopic eczema (Ferreira et al., 2017). Thus, we have validated that our model can identify relevant biomarker candidates and therapeutical targets also in the context of another immune-mediated disease, i.e., asthma.

## 4 Discussion

In the present study we have shown that simple mRNA-protein models, in which the protein expression is defined as a linear combination of the splice variants of a gene with a time delay accounting for the dynamical effect induced by post-transcriptional processes and protein synthesis, can improve our ability to predict protein abundance from mRNA expression. Furthermore, we demonstrated the impact that this finding can have within genome medicine by predicting and validating biomarkers for MS and asthma. Throughout the paper we aimed to increase the sensitivity in RNA-seq differential expression analysis. Sensitivity was measured using the fraction of nominally (*p* < 0.05) differentially expressed genes. This application revealed significantly more predicted biomarkers than by using off-the-shelf methods for RNA-seq data analysis only, which suggests increased sensitivity.

Despite being part of the central dogma and of uttermost importance in biology and medicine, the prediction of protein levels from mRNA levels has long been associated with low precision, which has been a matter of debate (Fortelny et al., 2017). Due to the complex process of mRNA-to-protein translation, there are several aspects that need to be considered (Liu et al., 2016). In this paper we thoroughly addressed two presumed main aspects; 1) how to incorporate splice variants into the prediction protein expression, and 2) how to deal with the time delay of the translation between mRNA and protein expression. Interestingly, both aspects were found to impact prediction of protein abundance, as shown in our combined model, although the incorporation of splice variants influenced the protein abundance prediction the most. Herein, we report splice variants to have a wider correlation profile, both positive and negative, than what would be expected, and our novel approach takes advantage of this anti-correlation between splice variants and proteins. In previous work, the impact of incorporating splice variants into protein predictions has been analysed. These studies have focused on mechanistic cell type independent factors such as splice variant-specific degradation rates (Eraslan et al., 2019). Instead, we found that the correlations were cell type-specific, and we constructed data-driven predictive models. To construct those models, we performed activation of NT_H_ cells followed by time-series analysis, which enabled us to infer the system based on its dynamics. A necessary requirement for such as model was dynamical data covering a decent number of time-points that allowed for the possibility of including modelling of intermediate time-points and the inference of time delays. However, the resulting Pearson correlations from our model need to be taken cautiously as we could not do a complete test as parts of the longitudinal data was visible to the model. From our models we proposed a biomarker discovery strategy which was validated in three steps. First, we found that usage of these models in complex disease enabled identification of more differentially expressed genes, which we therefore predicted as potential biomarkers. Second, we noted that many of the predicted proteins had previously been associated with MS and asthma, confirming that our strategy predicts relevant disease genes. Third, we validated one such protein as a biomarker in MS, namely sCD27. While sCD27 has already been associated with MS (van der Vuurst de Vries et al., 2017; Wong et al., 2018; Mahler et al., 2020), our clinical analysis of two independent cohorts yielded novel findings of remarkably good prognostic capabilities for treatment response and 4 years disease activity, which is important areas for early MS treatment selection.

Although incorporating splice variant information into the model was the main influential factor on the correlation, time delay also had an impact. The kinetics in translation of mRNA to protein is of general interest given its crucial importance in the design of experiments, for example in verifying relevance of mRNA expression to protein expression. Such models should ideally be functionally validated based on mechanistic principles, described by ordinary differential equations, such as the ones presented by for example Jovanovic et al. (2015). However, given that time-series experiments are time- and labor intensive, as well as expensive and predictive large-scale models are highly needed for biomarker discoveries, a database that provides the relevant time delay between mRNA expression and the expression of its corresponding protein would be immensely valuable. Here, we present such an atlas, comprising almost 5000 gene expression-to-protein translation kinetics (Supplementary Table S1).

A limitation with the paper is that we investigated few key cell types, namely T_H_1 cells, T_REG_ cells and B cells whereof wet lab experiments was only performed in one of these cell types. However, we were able to transfer the approach to two other cell type re-using data of other studies, demonstrating the robustness of the model assumptions. Furthermore, the chosen cell types are central in regulation of immune responses, and the T_H_ cells indeed are involved in many complex and common illnesses, like infectious, allergic, autoimmune and cardiovascular diseases and cancer (Farber, 2020).

In conclusion, we have constructed data-driven linear models incorporating splice variant information and time delay to predict protein expression from mRNA. We showed the general applicability of our approach by developing robust models for datasets from several cell types, and therefore the general principle of the model should be applicable to other cell types. For example, we expect this modelling strategy to be generally applicable to other cellular differentiation systems, such as embryonic stem cell differentiation, and to be increasingly useful for understanding basic biology and identification of new biomarkers as more RNA-seq and proteomic data sets become publicly available. Finally, we have shown that our proposed approach is of clinical relevance for prediction of validated biomarkers.

## Data Availability

The raw and processed RNA-seq data were submitted to the EMBL-EBI sequencing archive ArrayExpress and is available under the accession number E-MTAB-7775. The proteomics data were submitted to the EMBL-EBI proteomics repository PRIDE under the accession PXD013361. Pipeline and code for the mathematical modelling and bioinformatics analysis available from https://gitlab.com/Gustafsson-lab/splice_protein_predictions.
