# Supplementary material for: RNA-sequencing and mass-spectrometry proteomic time-series analysis of T-cell differentiation identified multiple splice variants models that predicted validated protein biomarkers in inflammatory diseases
Source: Front Mol Biosci. 2022 Aug 29;9:916128. doi: 10.3389/fmolb.2022.916128 (PMC9465313; doi:10.3389/fmolb.2022.916128)
Supplement: Supplementary file 7 [file DataSheet1.DOCX]

Supplementary Material

**RNA-sequencing and mass-spectrometry proteomic time-series analysis of T-cell differentiation identified multiple splice variants models that predicted validated protein biomarkers in inflammatory diseases**

Rasmus Magnusson^1^*, Olof Rundquist^1^*, Min Jung Kim^2^, Sandra Hellberg^3^, Chan Hyun Na^4^, Mikael Benson^5^, David Gomez-Cabrero^6^, Ingrid Kockum^7^, Jesper Tegnér^8,9,10^, Fredrik Piehl^7^, Maja Jagodic^7^, Johan Mellergård^11^, Claudio Altafini^12^, Jan Ernerudh^13^, Maria C. Jenmalm^3^, Colm E. Nestor^3^, Min-Sik Kim^14^ and Mika Gustafsson^1^

^1^Bioinformatics, Department of Physics, Chemistry and Biology, Linköping University, Linköping, Sweden.

^2^Department of Applied Chemistry, College of Applied Sciences, Kyung Hee University, Yong-in 446-701, Republic of Korea.

^3^Department of Biomedical and Clinical Sciences, Linköping University, Linköping, Sweden

^4^Department of Neurology, Institute for Cell Engineering, Johns Hopkins University School of Medicine, Baltimore, MD, USA

^5^Centre for Personalised Medicine, Linköping University, Linköping, Sweden.

^6^Navarrabiomed, Complejo Hospitalario de Navarra, Universidad Pública de Navarra, IdiSNA, 31008 Pamplona, Spain

^7^Department of Clinical Neuroscience, Center for Molecular Medicine, Karolinska Institute, 171 77, Stockholm, Sweden

^8^Biological and Environmental Sciences and Engineering Division, Computer, Electrical and Mathematical Sciences and Engineering Division, King Abdullah University of Science and Technology (KAUST), Thuwal 23955–6900, Saudi Arabia

^9^Unit of Computational Medicine, Department of Medicine, Solna, Center for Molecular

Medicine, Karolinska Institutet, Stockholm, Sweden.

^10^Science for Life Laboratory, Solna, Sweden.

^11^Department of Neurology, and Department of Biomedical and Clinical Sciences, Linköping University, Linköping, Sweden

^12^Department of Automatic Control, Linköping University, Linköping, Sweden

^13^Department of Clinical Immunology and Transfusion Medicine, and Department of Biomedical and Clinical Sciences , Linköping University, Linköping, Sweden

^14^Department of New Biology, Daegu Gyeongbuk Institute of Science and Technology, Daegu 711-873, Republic of Korea

*These authors contributed equally to this work and should be regarded as shared first authors.

Correspondence: mika.gustafsson@liu and maria.jenmalm@liu.se

***Correspondence:**Mika Gustafsson Maria Jenmalm
[mika.gustafsson@liu.se](mailto:mika.gustafsson@liu.se) [maria.jenmalm@liu.se](mailto:maria.jenmalm@liu.se)

# Supplementary Data

**Optimization of time series analysis for RNA-seq**

To establish the earliest informative time point for the upcoming RNA-Seq, naive CD45RA^+^ CD4^+^ T-cells were isolated and differentiated as described in the materials and methods with sampling at 3, 5, 10, 15, 30 and 60 minutes. At each sampling point, samples were washed twice with PBS by centrifugation at 1000g for 1 minute followed by snap-freezing in a dry-ice ethanol bath and transferee to -80°֯C for storage. RNA was then extracted with a RNeasy mini kit (Qiagen,Hilden, Germany) and first strand cDNA synthesis performed with TaqMan™ Reverse Transcription Reagents. The cDNA was then mixed with TaqMan™ Fast Advanced Master Mix and FAM labelled TaqMan probes and primers. The qPCR assay was carried out on a 7900HT Fast Real-Time PCR System instrument (Applied Biosystems, Waltham, MA, USA) in 20 µl on a 96 well plate with probes for *IL2*, *IFNG*, *TBX21*, *GUSB* and ACTB. GUSB and ACTB were used as reference genes. All cDNA and TaqMan reagents were purchased from Applied Biosystems. All probes and primers were purchased from ThermoFisher Scientific (Waltham, MA, USA). qPCR was performed in technical triplicates for each time point and probe and the experiment was repeated six times (one repeat lacked the time points for 3 and 10 minutes). Based on this the earliest time point for the RNA-seq was set to 30 minutes.

# Supplementary Figures


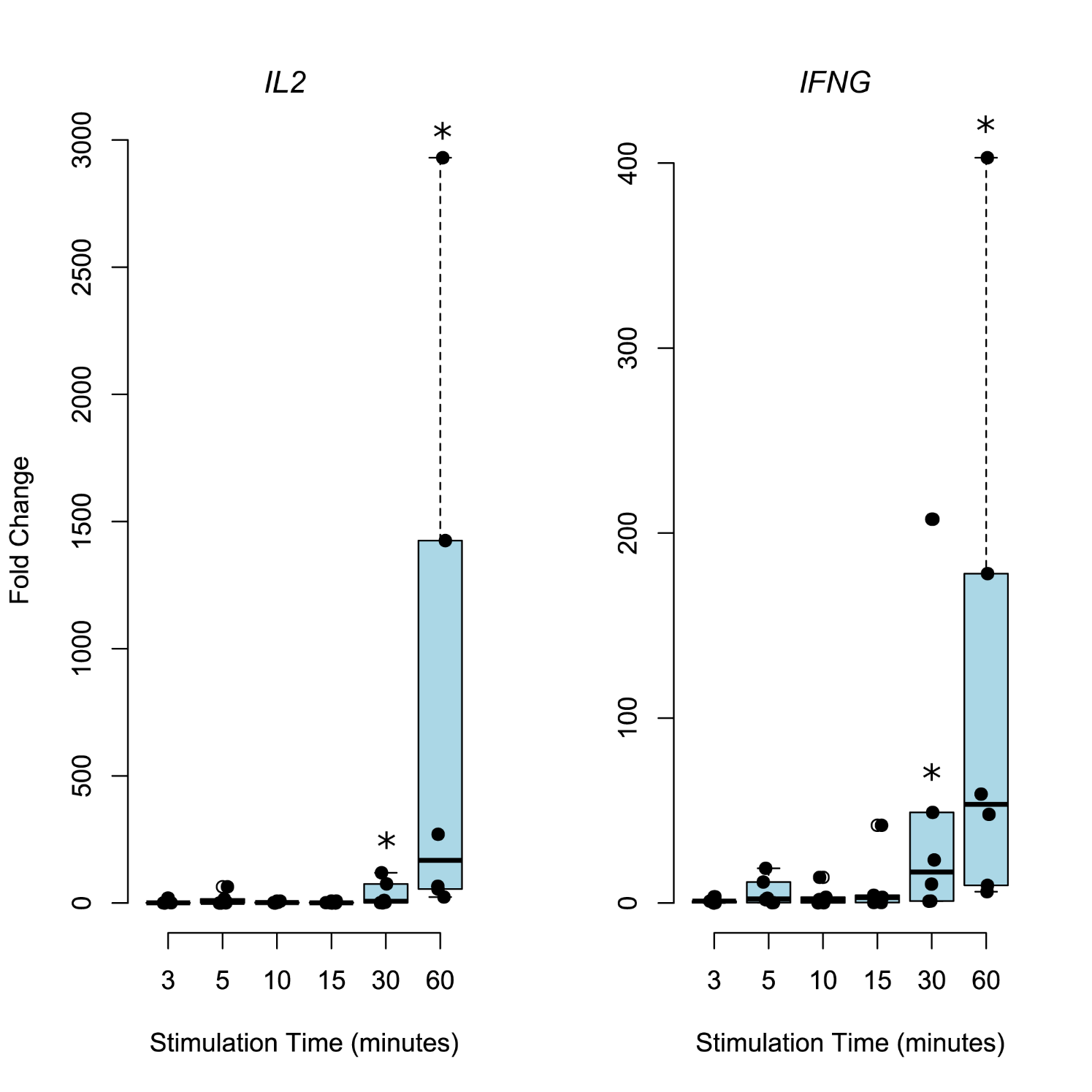


**Figure S1.** In vitro T_H_1 differentiation from naïve T cells resulted in significant consistent upregulation of *IL2* and *IFNG* expression observed at 30 minutes and at 60 minutes (p<0.05, student’s t-test) as measured by qRT-PCR.


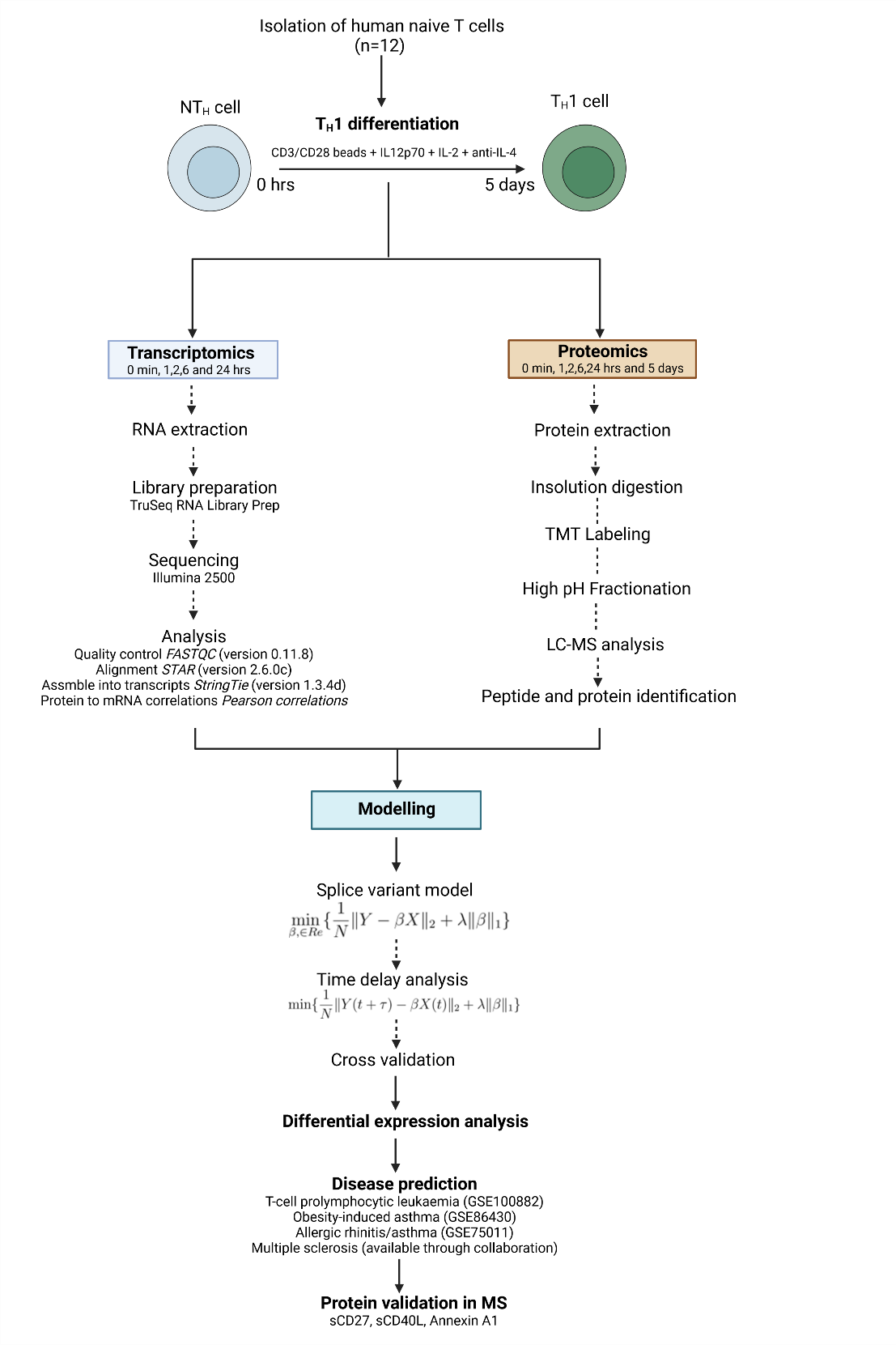


**Figure S2.** Overview of the study. More details regarding materials and methods can be found in the article.


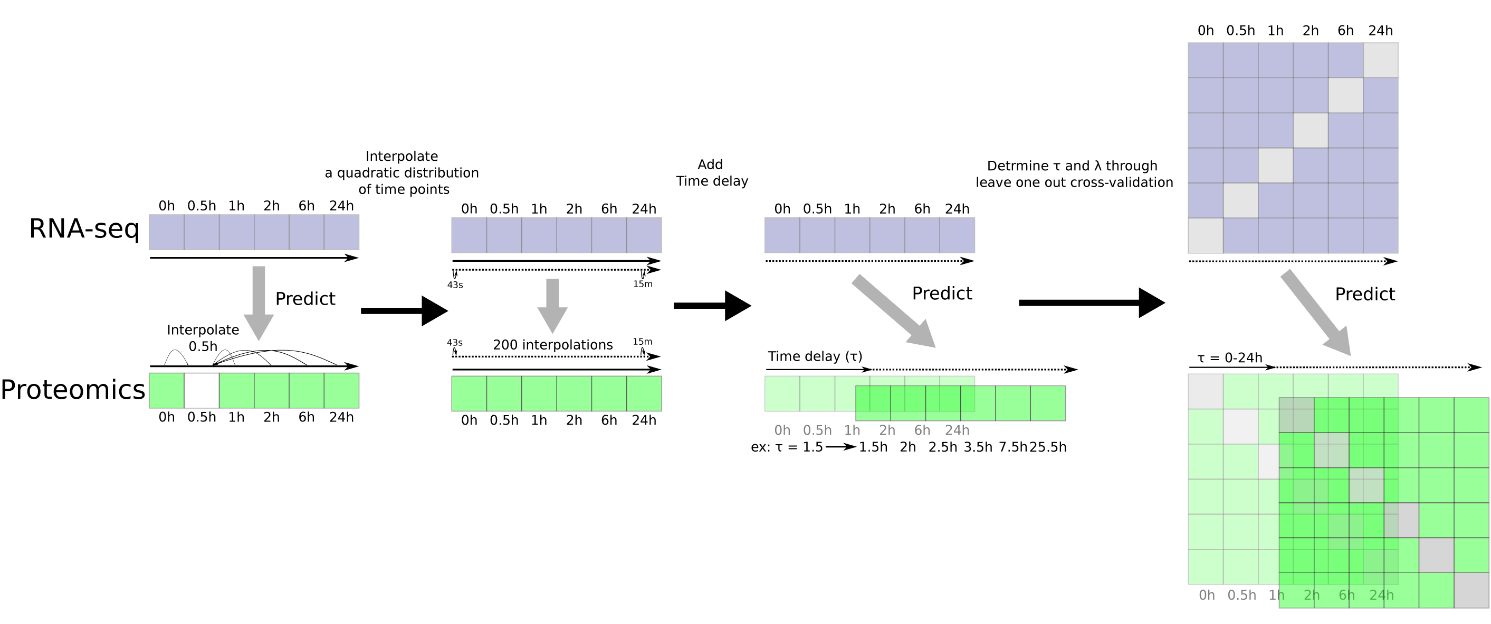


**Figure S3. Model selection and cross validation scheme.** A graphical representation of the model selection and cross validation procedure. In brief, first the 0.5h time point was interpolated for the proteomics and a L1-penalized linear regression model was applied. Time delay was then added to the model by first interpolating a quadratic distribution of time points such that the distance between interpolated time points was 43s to 15 minutes followed by the addition of a general protein time delay, τ. Finally, the optimal values for τ and λ were selected for each protein by using a leave on out cross validation.

**
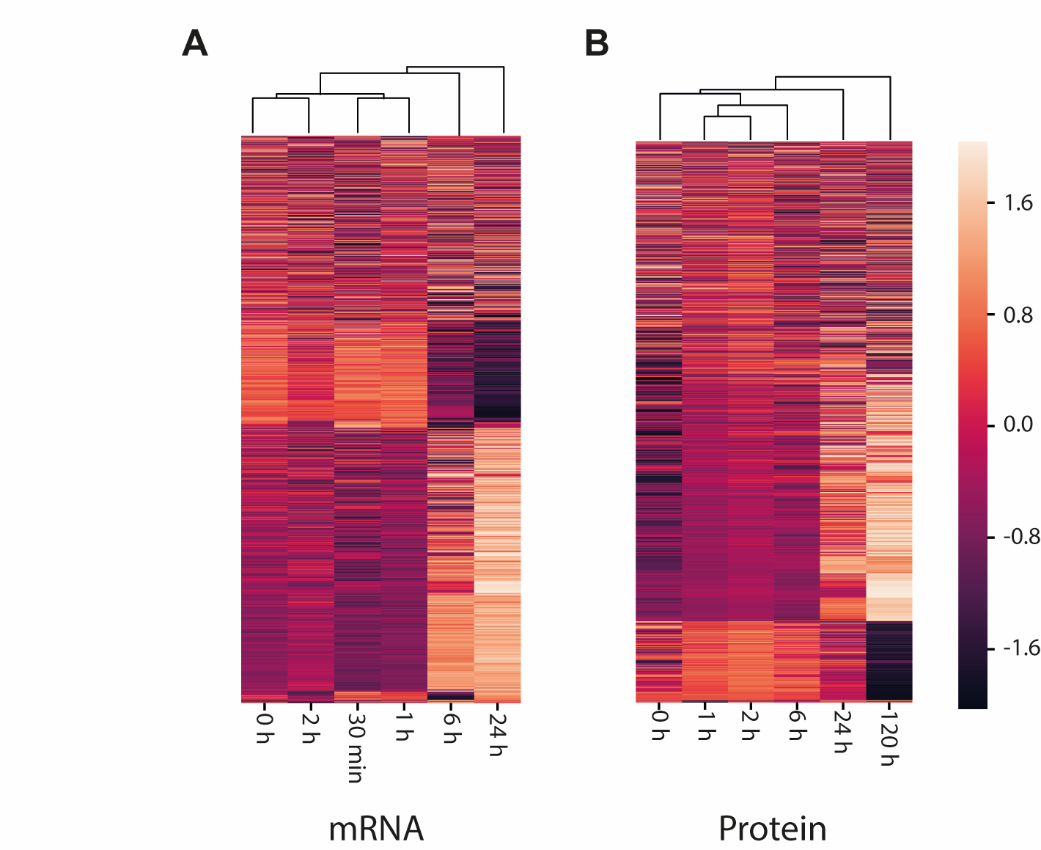
Figure S4.** Hierarchical clustering of time-series **(A)** RNA-seq and **(B)** mass-spectrometry proteomics data of T_H_1 differentiation showed that samples clustered well with respect to time. We analysed the data quality, and the similarities of the different measured time points, by hierarchical clustering. For all 4,860 genes and corresponding protein time series that were used for the model estimation, we performed a Z-normalization of each time series and performed a hierarchical clustering using the Python package Scipy. We used the default parameters ‘single linkage’, minimizing the Euclidian distance between clusters. In (A), we clustered the mRNA gene expressions and found all biological repeats except the time point t=2h to cluster with their closest neighbours in time. In (B), we saw a perfect clustering of the time point in a consecutive order. The clustering in (A) and (B) show that there is a distinguishable biological signal of activation throughout both the mRNA expression and protein abundance datasets.


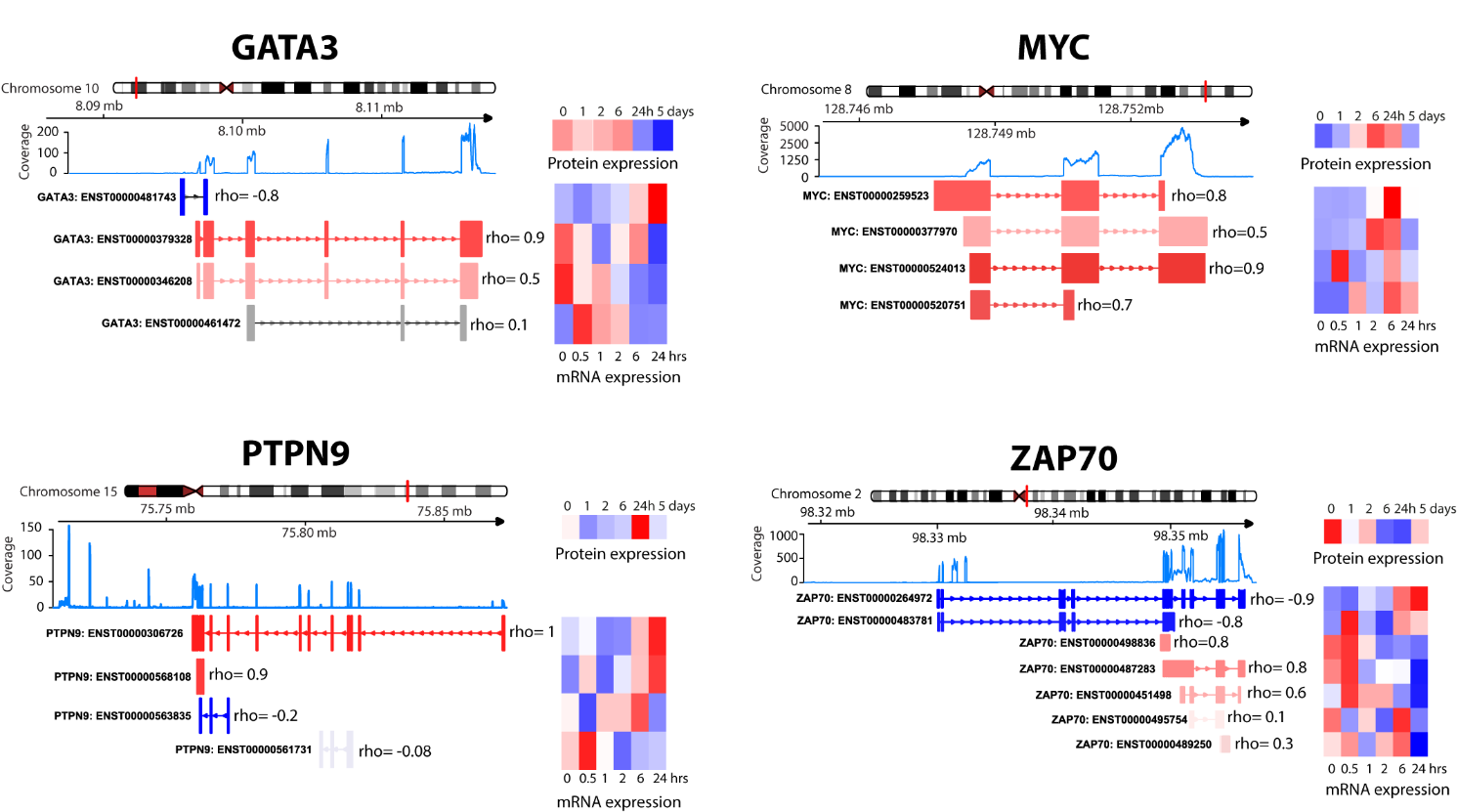


**Figure S5.** Additional examples of genes with multiple correlating and anti-correlating transcripts: GATA3, MYC, PTPN9 and ZAP70 are all examples of genes that has both correlating and anti-correlating transcripts. Each transcript is colour coded according to their Pearson correlation between mRNA and protein expression. Relative mRNA and protein expression is depicted as heatmaps on the right and the average coverage of reads over exons is displayed as blue line plot in the middle.

# Supplementary Tables

**Table S1: Table of all genes with their transcripts and modelling coefficients across all three models.** Each sheet represents one cell-type, human T_H_1, T_REG_, and mice B-cells, where each row represent one transcript to protein association (see section time-delayed models for details). Column A is the gene name of the corresponding protein, column B the splice variant name, column C the value of the linear coefficient, column D is the correlation between measured and predicted protein abundance values, column E is the corresponding time-delay (τ).

**Table S2: Patient and healthy control characteristics at inclusion and follow-up for the CIS and newly diagnosed MS cohort and the pre- and post-treatment cohort.**

**Table S3: Top 20 differentially expressed proteins in multiple sclerosis.** Markers previously associated with multiple sclerosis are indicated in grey. The study included nine healthy controls, six relapse remitting and nine secondary progressive MS patients.

**Table S4. sCD27 levels measured by ELISA.**

**Table S5: Top 20 differentially expressed proteins in asthma.** Markers previously associated with asthma are indicated in grey. The study included 15 healthy controls and 40 asthmatic patients.

**Table S6: Predicted differentially expressed proteins in asthma not detectable by standard RNA-seq data analysis.**
